# Identifying key descriptors in surface binding: interplay of surface anchoring and intermolecular interactions for carboxylates on Au(110)†
†Electronic supplementary information (ESI) available: Supporting experimental methods and supporting discussion are included in the supplementary information. See DOI: 10.1039/c7sc05313d


**DOI:** 10.1039/c7sc05313d

**Published:** 2018-03-12

**Authors:** Christopher R. O'Connor, Fanny Hiebel, Wei Chen, Efthimios Kaxiras, Robert J. Madix, Cynthia M. Friend

**Affiliations:** a Department of Chemistry and Chemical Biology , Harvard University , Cambridge , MA 02138 , USA . Email: friend@fas.harvard.edu; b School of Engineering and Applied Sciences , Harvard University , Cambridge , MA 02138 , USA; c Department of Physics , Harvard University , Cambridge , MA 02138 , USA

## Abstract

The relative stability of carboxylates on Au(110) was investigated as part of a comprehensive study of adsorbate binding on Group IB metals that can be used to predict and understand how to control reactivity in heterogeneous catalysis. The binding efficacy of carboxylates is only weakly dependent on alkyl chain length for relatively short-chain molecules, as demonstrated using quantitative temperature-programmed reaction spectroscopy. Corresponding density functional theory (DFT) calculations demonstrated that the bidentate anchoring geometry is rigid and restricts the amount of additional stabilization through adsorbate-surface van der Waals (vdW) interactions which control stability for alkoxides. A combination of scanning tunneling microscopy (STM) and low-energy electron diffraction (LEED) shows that carboxylates form dense local islands on Au(110). Complementary DFT calculations demonstrate that adsorbate–adsorbate interactions provide additional stabilization that increases as a function of alkyl chain length for C_2_ and C_3_ carboxylates. Hence, overall stability is generally a function of the anchoring group to the surface and the inter-adsorbate interaction. This study demonstrates the importance of these two important factors in describing binding of key catalytic intermediates.

## Abstract 2

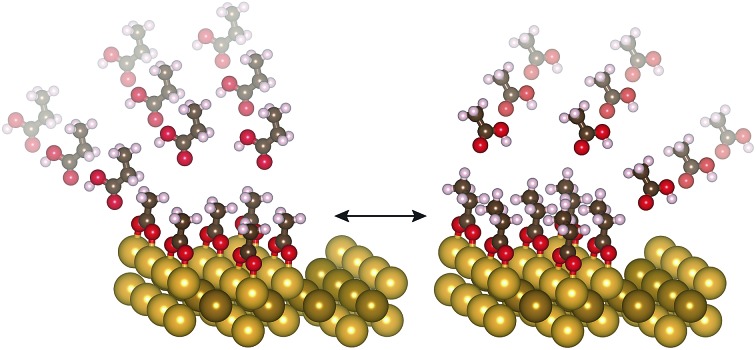
The relative stability of carboxylates on Au(110) was investigated as part of a comprehensive study of adsorbate binding on Group IB metals that can be used to predict and understand how to control reactivity in heterogeneous catalysis.

## Introduction

Heterogeneous catalysis is key to ensuring sustainability in chemical transformations.^1^ Accordingly, there is a drive to develop principles for designing efficient catalytic processes. One approach to establishing such principles is the creation of a database of key properties (“descriptors”) that can be related to catalytic performance;^2^–^6^ for example, binding energies of key intermediates to specific materials. While the so-called Materials Genome Initiative^7^–^9^ seeks a limited set of key descriptors for complex materials design processes, it is important to understand the underlying factors that contribute to descriptors, such as binding energy.

Carboxylate species are an important class of molecules that are both reactive intermediates and also potential poisons in oxidative processes. For example, carboxylates are intermediates in the oxidation of alcohols and olefins, yielding both carboxylic acids and CO_2_, and electrochemical reduction of CO_2_.^10^–^27^ Carboxylate intermediates also strongly bind to surfaces so as to block sites.^17^,^22^–^24^ For example, carboxylates formed in over-oxidation of alcohols block subsequent formation of the key alkoxide intermediates; thus, suppressing activity.^17^ Because of the broad importance of carboxylates in oxidation catalysis, we have systematically investigated them to develop a hierarchy of binding strength and also to provide a more detailed understanding of the factors that dictate their stability. This investigation is part of the development of a database for key intermediates on Group IB metals (Cu, Ag and Au), following on our prior studies of alkoxides.^28^–^33^ Herein, binding of carboxylates to Au(110) is investigated because of the broad interest in it as a selective oxidation catalyst.^34^–^40^

Creation of carboxylates on Au(110) is facile because organic acids generally react with adsorbed oxygen atoms^15^,^41^,^42^ by an acid–base mechanism as illustrated for carboxylic acids:

1 2RCOOH_(g)_ + O_(a)_ → 2RCOO_(a)_ + H_2_O_(g)_

The carboxylic acids do not react with clean metallic Au; thus, the surface concentration of the carboxylates formed is entirely controlled by the initial coverage of adsorbed oxygen and all oxygen can be removed as water to solely produce the carboxylate species at a specific surface coverage.^41^,^42^ Furthermore, carboxylates adsorbed on Au are also proton acceptors, so that they can react with other gas phase acids exposed to the surface, providing a means of evaluating the relative binding strength of various carboxylates. Indeed, the relative gas phase acidities of reactants qualitatively predict the stability of reaction intermediates.^28^

2 R′COOH_(g)_ + RCOO_(a)_ → R′COO_(a)_ + RCOOH_(g)_

This competition can be described by a series of steps, the first of which is known from measured gas-phase acidities:

3 RCOO′H_(g)_ + RCOO_(g)_^–^ → R′COO_(g)_^–^ + RCOOH_(g)_, Δ(Δ*H*_acid_)

4 R′COO_(g)_^–^ → R′COO_(a)_, Δ*H*_ads_(R′COO_(g)_^–^)

5 RCOO_(a)_ → RCOO_(g)_^–^, –Δ*H*_ads_(RCOO_(g)_^–^)

It follows that gas phase acidity is an accurate predictor of the competitive binding only if the energetic difference in bonding of the adsorbed conjugate bases to the surface is negligible and that adsorbate bonding is effectively ionic due to the anionic nature of adsorbates.^28^

Prior studies demonstrated that acetate forms condensed islands on Au(110) due to net attractive interactions between adsorbed species.^43^ Hence, these intermolecular interactions must be considered along with surface-adsorbate binding in evaluating overall stability of the carboxylates. Carboxylates, including acetate and formate, are more strongly bound than their alkoxide counterparts, ethoxy and methoxy, on Au, raising questions regarding the factors that contribute to this stronger binding. The primary anchoring bond, surface structure, intermolecular interactions and noncovalent interactions between the surface and the pendant alkyl group can all contribute to overall binding.

In this study the experimental hierarchy of binding stability for saturated carboxylates on Au(110) was determined through a series of adsorbate displacement experiments. Saturated carboxylates have a similar stability with only a small stabilization associated with longer chains. DFT calculations demonstrate that the bidentate geometry causes rigid binding which restricts further stabilization through adsorbate-surface van der Waals (vdW) interactions. Scanning tunneling microscopy (STM) and low-energy electron diffraction studies show that carboxylates form dense local islands; further, DFT calculations demonstrate that adsorbate–adsorbate vdW interactions play a pivotal role in determining carboxylate stability by increasing stabilization for longer chain carboxylates. Hence, this study refines the understanding of vdW interactions in determining the stability of intermediates.

## Methods

### Experimental

Temperature-programmed reaction spectroscopy and low-energy electron diffraction (LEED) experiments were performed under ultrahigh vacuum conditions in a chamber with a base pressure < 3.0 × 10^–10^ Torr. Temperature programmed experiments were performed using a triple filter Hiden quadrupole mass spectrometer (QMS, HAL-Hiden/3F). During all temperature programmed experiments, a –100 V bias was applied to the sample to prevent possible electron-stimulated reactions. A heating rate of 5 K s^–1^ was used for all experiments. LEED experiments were performed using Perkin-Elmer Phi Model 15-120 LEED Optics. In a separate chamber, scanning tunneling microscopy was performed with an Omicron VT-STM under ultra-high vacuum of base pressure *P* < 1.0 × 10^–10^ mbar, using commercial mechanically cut PtIr tips purchased from Veeco.

Separate Au(110) crystals were prepared for the reactivity^14^ and STM^35^ measurements according to procedures described previously. Controlled amounts of atomic oxygen were deposited using ozone in established procedures.^17^,^41^ For STM experiments, the coverage of adsorbed oxygen was calculated by counting the oxygen atoms in zigzag chains and the top layer gold atoms in a given area; for temperature programmed experiments, the coverage of adsorbed oxygen was calibrated by the integrated O_2_ signal due to atomic oxygen recombination above 500 K for the saturation coverage of 1 ML.^44^

Experiments were performed on Au(110) covered with 0.05 ML adsorbed atomic oxygen, hereafter referred to as O/Au(110). The liquid organics were purified according to procedures described previously and the gas phase vapor was leaked in the chamber while monitoring the rise in pressure of the chamber.^31^,^32^ Temperature programmed experiments were performed with each reactant to identify its signature products and displacement reactions were preformed to determine the relative binding stability of selected pairs of carboxylates as described in the (ESI†). The quantitative analysis of the temperature programmed reaction data was performed as described in the ESI.†

### Computational details

DFT calculations were performed using the Vienna *ab initio* simulation package (VASP).^45^ The projector augmented wave (PAW) method^46^ was used with a plane-wave basis set (kinetic energy cutoff 400 eV) and the PBE exchange-correlation functional.^47^ Dispersion interactions were approximated with the Tkatchenko–Scheffler method.^48^

The Au(110) supercell was built out of 5 atomic layers with the two bottom layers constrained to their bulk positions and more than 10 Å of vacuum layer above the adsorbates. The bulk positions were adapted depending on whether dispersion interactions were included or not (lattice constant of 4.11 Å with and 4.16 Å without dispersion correction, which is close to the reported experimental value 4.08 Å).^49^ Laterally, a 4 × 4 (4 × 2) periodicity with respect to the Au(110)-(1 × 1) surface was used, with a 3 × 7 × 1 (7 × 7 × 1) Gamma-centered *k*-point mesh when isolated molecules (initial adsorption geometry exploration, namely top, bidentate top, bidentate bridge and chelating, and dense layers) were considered. Unconstrained atoms were relaxed to a force threshold of 0.01 eV Å^–1^.

## Results and discussion

### Displacement reactions to determine the relative stability of the carboxylates

Reaction products and their peak temperatures were first determined for each of the carboxylates studied—propanoate, trifluoroacetate, acetate and formate. All the carboxylates produce CO_2_; however, there are other signature products at different temperatures (Table 1), enabling the determination of the amounts of specific carboxylates present based on the temperature and magnitude of signature product evolution. In all cases, the signature products and the peak temperatures were determined using temperature programmed reaction after exposure of 0.05 ML of atomic oxygen on Au(110) to an excess of the carboxylic acid at 300 K, yielding 0.10 ML of the carboxylate. All adsorbed atomic oxygen is removed as water under these conditions. The coverage was held fixed to avoid coverage-dependent variation in the temperature for product evolution. The methodology is exemplified here by acetic acid/trifluoroacetic acid pair; data for other acid pairs are provided in the ESI (Fig. S1–S3; Table S1†).

**Table 1:** Characteristic reactions for adsorbed carboxylates on Au(110)

Organic acid (adsorbed carboxylate)	Characteristic reaction products	Product peak temperature (K)
CH_3_CH_2_COOH (CH_3_CH_2_COO_(a)_)	CH_2_CH_2_, CO_2_	550
CF_3_COOH (CF_3_COO_(a)_)	CF_3_, CO_2_	590
CH_3_COOH (CH_3_COO_(a)_)	CH_3_, CO_2_	580
HCOOH (HCOO_(a)_)	HCOOH, CO_2_	350

**Fig. 1:**
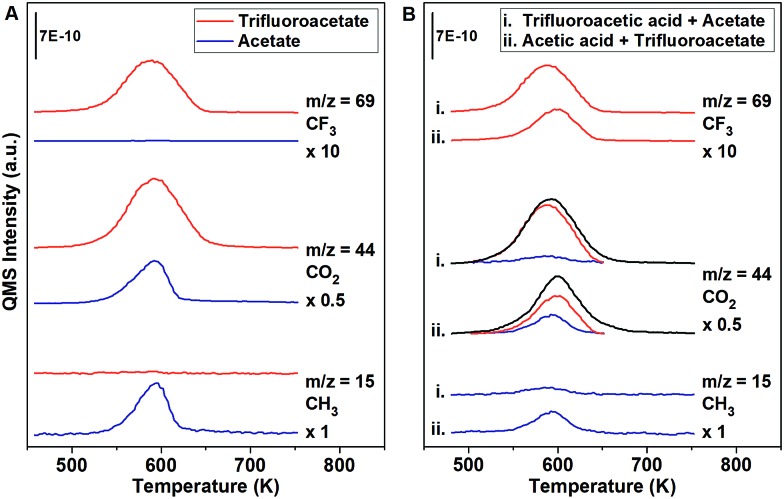
Temperature programmed experiments show nearly complete displacement of acetate by trifluoroacetic acid on Au(110) at 300 K. (A) The characteristic products for reaction of 0.10 ML of isolated trifluoroacetate (red) decomposed to CF_3_ and CO_2_ at 590 K, while 0.10 ML of isolated acetate (blue) decomposed to CO_2_ and CH_3_ at 580 K. (B) The introduction of (i) excess trifluoroacetic acid to acetate and (ii) excess acetic acid to trifluoroacetate yields products characteristic of a majority species trifluoroacetate and a minority species acetate. The deconvolution of the CO_2_ peak for acetate (blue) and trifluoroacetate (red) is determined by using a selectivity fraction on the CH_3_ and CF_3_ peaks. Both orders of adsorption show displacement that favors trifluoroacetate.

A pure layer of acetate (0.1 ML) decomposed to CO_2_ (*m*/*z* = 44) and CH_3_ (*m*/*z* = 15) at 580 K (Fig. 1A, blue). In separate experiments, trifluoroacetate (0.1 ML) decomposed to CO_2_ (*m*/*z* = 44) and CF_3_ (*m*/*z* = 69) at 590 K (Fig. 1A, red). The differences in the temperatures for decomposition and the differences in the signature products provides the basis for the quantitative determination of the amounts of trifluoroacetate and acetate in the displacement experiments. Since acetate and trifluoroacetate yield CO_2_ (*m*/*z* = 44) at overlapping temperatures, the unique evolution of CF_3_ (*m*/*z* = 69) from adsorbed trifluoroacetate at 590 K and CH_3_ (*m*/*z* = 15) from acetate at 580 K were used to quantify the relative amounts of adsorbed trifluoroacetate or acetate, respectively (eqn (6)–(8)).

6 CF_3_COOH + CH_3_COO_(a)_ ⇌ CF_3_COO_(a)_ + CH_3_COOH

7 CH_3_COO_(a)_ → CH_3(g)_ + CO_2(g)_

8 CF_3_COO_(a)_ → CF_3(g)_ + CO_2(g)_

Nearly complete displacement of acetate by trifluoroacetate is demonstrated by performing competition experiments (Fig. 1B), demonstrating that trifluoroacetate has a distinctly larger binding energy to the surface than acetate. By performing the experiments in both orders of adsorption, kinetic factors are ruled out. The exposure of excess trifluoroacetic acid to 0.10 ML of adsorbed acetate leads to nearly complete displacement, based on the predominance of CF_3_ evolution at 590 K and the absence of CH_3_ at 580 K (Fig. 1B, i). The exposure of excess acetic acid to 0.10 ML of adsorbed trifluoroacetate leads to partial displacement as evidenced by the evolution of both CF_3_ at 590 K and CH_3_ at 580 K (Fig. 1B, ii). The nearly complete displacement of acetate by trifluoroacetate when exposed to trifluoroacetic acid and limited displacement of trifluoroacetate by acetate when exposed to acetic acid clearly demonstrates that trifluoroacetate is more stable than acetate. The effect was observed for both orders of displacement, showing that this is a thermodynamic, not a kinetic, effect.

The selectivity of the decomposition of trifluoroacetate and acetate was determined to be constant up to 0.10 ML of carboxylate, within experimental error, so CF_3_ and CH_3_ can be used to quantify the presence of trifluoroacetate and acetate, respectively. The deconvolution of the measured CO_2_ peak is performed for acetate and trifluoroacetate by using the measured CH_3_ and CF_3_ signals according to the procedure described in the ESI.†

A hierarchy of binding efficacy was established using this displacement method (Table 2). Generally, longer alkyl chain lengths lead to somewhat stronger binding, which is qualitatively similar to alkoxide binding.^30^–^32^ Likewise, the stabilities of the carboxylates generally increase with the gas phase acidity of their conjugate acid (Table 2), with trifluoroacetic acid being the exception. Specifically, the gas phase acidities of trifluoroacetic acid (Δ*H*_acid_ = 1351 KJ mol^–1^) and propanoic acid (Δ*H*_acid_ = 1454 KJ mol^–1^) indicate that trifluoroacetate should bind much more strongly than propanoate, which is not in agreement with the experiments. A similar effect of fluorination was observed in the relative stabilities of trifluoroethoxy *vs.* ethoxy and propoxy on Au(111) and was attributed to repulsive interactions between the fluorine atoms and the surface, that decreased the heat of adsorption of the trifluoroethoxy relative to what is expected based on gas phase acidity.^30^ Nonetheless, there appears to be a general trend between the chemical structure of carboxylates and the relative binding stability of the carboxylates, consistent with previous studies on silver,^28^ gold,^30^,^31^ copper^32^ and anatase TiO_2_.^51^

**Table 2:** The ordered stabilities of surface carboxylate intermediates, the gas phase acidity of their parent acid, the reactions used to test their relative stabilities, and the equilibrium constants relative to acetate

	Conjugate base	Gas phase acidity^*a*^ (KJ mol^–1^)	Probe reaction	Exp. K^*b*^
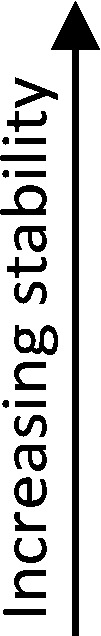	Propanoate	1454 ± 12	CH_3_COO_(a)_ + CH_3_CH_2_COOH → CH_3_COOH + CH_3_CH_2_COO_(a)_	4

	Trifluoroacetate	1351 ± 17	CF_3_COO_(a)_ + CH_3_CH_2_COOH → CF_3_COOH + CH_3_CH_2_COO_(a)_	2

			CH_3_COO_(a)_ + CF_3_COOH → CH_3_COOH + CF_3_COO_(a)_	2

	Acetate	1459 ± 9	CH_3_COO_(a)_ + CH_3_COOH ↔ CH_3_COOH + CH_3_COO_(a)_	1

	Formate	1444 ± 12	CH_3_COO_(a)_ + HCOOH ← CH_3_COOH + HCOO_(a)_	0.9

^*a*^Gas phase acidity (taken from the NIST database)^50^ is defined as Δ*H* for BH_(g)_ → B_(g)_^–^ + H_(g)_^+^ (KJ mol^–1^).

^*b*^Equilibrium constant is determined at 260 K for formate/acetate and 300 K for other pairs.

If the difference of enthalpies of adsorption between the two carboxylate anions could be determined, the equilibrium constant for the competition of two species could be determined quantitatively from the gas phase acidity difference (assuming their entropies of adsorption are nearly equal). In fact, if the entropies and energies of adsorption of the ions are nearly the same, the gas phase acidity can be used as a qualitative evaluation of relative binding stability; however, this is not always the case, and, though the gas phase acidity may give the trends in stability, it cannot be expected to yield accurate values for the equilibrium constants determined here. The difference of entropic contributions of adsorption can be empirically approximated^52^ but the energy of adsorption of the gas phase anions cannot be determined accurately, so the gas phase acidity can serve only as a guide for the relative binding stability. Exceptions may occur.

### Determination of equilibrium constants

To quantitatively evaluate the relative binding of various carboxylates, it is necessary to determine the equilibrium constants (eqn (2)) for competitive binding. To this end, a new method for determining equilibrium constants that uses sequential dosing of different carboxylic acids was developed that probes the kinetics of the displacement reactions to determine the equilibrium constant (Table 2). This method was required because introducing a gaseous mixture of two carboxylic acids resulted in undesirable side reactions.

Briefly, if R′COOH is exposed to the surface above its desorption temperature but below the temperature at which RCOO_(a)_ decomposes, the rate of displacement of RCOO_(a)_ is:9
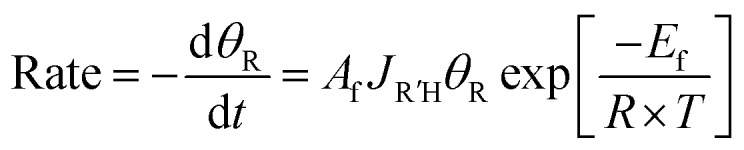
where *J*_R′H_ is the flux of R′COOH_(g)_ (ML s^–1^), *A*_f_ is the pre-exponential factor for the forward reaction (ML^–1^), *θ*_R_ is the coverage of RCOO_(a)_ (ML), *E*_f_ is the activation energy of the forward displacement reaction (J mol^–1^), *R* is the gas constant (J mol^–1^ K^–1^), and *T* is the temperature of the sample (K). For a controlled molecular flux, exposure time (*t*_f_) and initial coverage of carboxylate probed by both the forward and reverse displacement reaction, the equilibrium constant can be expressed as:10
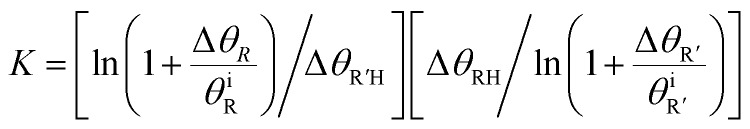
where Δ*θ*_R_ is the change in RCOO_(a)_ coverage (ML), *θ*iR is the initial coverage of RCOO_(a)_ (ML), Δ*θ*_R′H_ is the dosage of R′COOH onto the surface (ML), Δ*θ*_R′_ is change in R′COO_(a)_ coverage (ML), *θ*iR′ is the initial coverage of R′COO_(*a*)_ (ML), Δ*θ*_RH_ is the coverage of RCOOH exposed to the surface (ML). The derivation of this equation is detailed in the ESI.†

The equilibrium constants for various pairs of carboxylic acids were determined using this method (Table 2), as described in detail in the ESI.† The validity of the kinetic model used was established by accurately predicting the relative surface concentration of acetate and propanoate resulting from a well-defined increase in the acetic acid exposure to adsorbed propanoate (Fig. S4†). These results establish a quantitative basis for evaluating the relative binding efficacies of different carboxylates.

As the length of the alkyl chain increases, the surface stability of the carboxylate slightly increases, as demonstrated by the series of formate, acetate and propanoate (Table 2). Note that the reliability of the equilibrium constants is confirmed by the consistency among the measurements made for the competitions involving trifluoroacetic, acetic and propanoic acid. A similar, but stronger, dependence has been reported for adsorbed alkoxide species on gold surfaces^30^,^31^ that was explained by increasing adsorbate-surface vdW interactions as a function of alkyl chain length. The stronger dependence of binding strength on alkyl chain length for alkoxides is illustrated by comparison of the equilibrium constant for the methanol/ethanol competition, which is 5 in favor of ethoxy binding on Au(110),^31^ compared to the equilibrium constant of ∼1 for the formic acid/acetic acid pair measured here. A similarly weaker dependence for the carboxylates of increasing chain length relative to analogous alkoxide pairs is measured in all cases. A key question is why there is a weaker dependence on alkyl chain length for the carboxylates.

### Carboxylate adsorbate-surface interactions

**Fig. 2:**
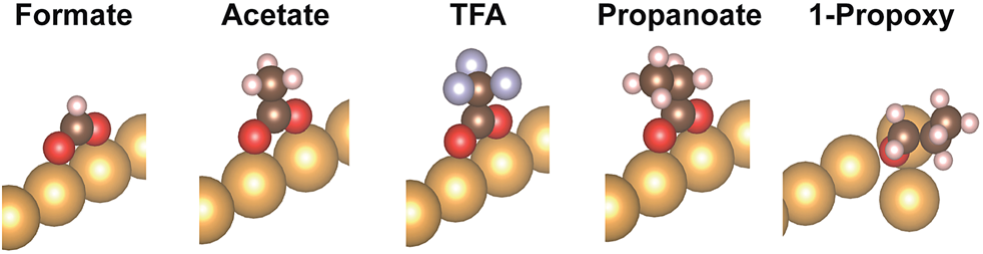
Adsorption geometry of the carboxylates formate, acetate, trifluoroacetate (TFA), propanoate, and of the alkoxy 1-propoxy as a reference. All carboxylates adopt a bidentate top geometry. Only the Au atoms of the top row of the missing row Au(110)-(1 × 2) reconstructed surface are depicted.

DFT calculations that include vdW corrections were performed to elucidate the origin of the relative stability of isolated carboxylates on Au(110) including formate, acetate, trifluoroacetate and propanoate (Fig. 2). Prior studies of alkoxides on Au and Cu demonstrated the importance of including vdW corrections. The anchoring of acetate on Au(110) was previously investigated in detail^43^ and the bidentate top configuration was significantly more stable than either the monodentate or bridge configurations. The same anchoring configuration was obtained for other carboxylates in the homologous series (Fig. S5, S6†). Adsorption within the troughs was also tested for propanoate and found to be less stable than the bridging configuration (Fig. S7†). The stability of isolated carboxylates was quantified according to the reaction:11
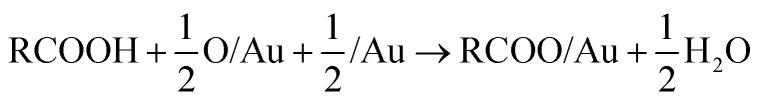
where /Au represents the bare Au supercell and *e.g.* O/Au represents the O adsorbed supercell. The calculated binding hierarchy for isolated carboxylates varies only slightly across the series (Table 3) and only partially agrees with experiments even if vdW corrections are included, suggesting additional effects. Whether vdW corrections are included or not (PBE *vs.* PBE + vdW), the binding of formate, acetate and propanoate are all essentially the same and trifluoroacetate is only slightly more strongly bound. In contrast, the binding energies of alkoxides increases by 0.1 eV for every CH_2_ group added.^31^ Furthermore, the energy gained from inclusion of vdW interactions is small for the isolated carboxylates in comparison to the corresponding alkoxides on the same surface. For example, the increase in energy for propanoate is ∼0.2 eV when vdW corrections are included, compared to an increase of ∼0.4 eV for 1-propoxy^31^ (Table 3).

**Table 3:** Reaction energy (*E*(rxn)) per adsorbate calculated for each carboxylate adsorbed onto a 4 × 4 × 1 slab of Au(110)-(1 × 2) surface structure using the PBE functional (PBE), and the vdW-corrected PBE (PBE + vdW) according to eqn (11)^*a*^

	*E*(rxn) (eV)	
	(PBE)	(PBE + vdw)
Propanoate	–0.85	–1.04
Trifluoroacetate	–0.97	–1.14
Acetate	–0.82	–0.99
Formate	–0.89	–1.01
1-Propoxy	–0.22	–0.64

^*a*^The reaction energy of 1-propoxy was included as a reference to demonstrate the stronger effect of vdW interactions for alkoxides.

These differences are the consequence of differences in the binding to the surface of the carboxylates *vs.* the alkoxides (Table 4). The computed geometries of the carboxylates reveal that (1) methyl-surface distances are much larger than for alkoxides (they do not vary much among the carboxylates), and (2) at most small geometry changes are induced by vdW contributions. The possible molecular rotations bringing the carbon groups closer to the surface have been evaluated in detail (Fig S8; Table S2†). The robustness of the bidentate top adsorption geometry is so strong that any stabilization from increased adsorbate-surface vdW interactions is counterbalanced by energy loss from non-optimal anchoring geometry.

**Table 4:** Geometrical^*a*^ characteristics of the carboxylates investigated, with and without vdW contributions

		*h*_C_0__ (Å)	*h*_C_1__ (Å)	*h*_C_2__ (Å)	C_0_C_1_C_2_ angle (°)
Propanoate	(PBE)	2.74	4.26	4.94	115.4
	(PBE + vdW)	2.74	4.26	4.94	115.5
Trifluoroacetate	(PBE)	2.74	4.31		
	(PBE + vdW)	2.74	4.30		
Acetate	(PBE)	2.74	4.25		
	(PBE + vdW)	2.74	4.25		
Formate	(PBE)	2.73			
	(PBE + vdW)	2.72			
1-Propoxy^*b*^	(PBE)	2.77	3.38	4.91	112.3
	(PBE + vdW)	2.79	3.26	4.78	111.9

^*a*^ *h*_C*_n_*_ is the methyl group-surface distance for the *n*th group, starting from the carboxyl group C_0_.

^*b*^1-Propoxy heights are given with respect to the (111) microfacet.

### Carboxylate adsorbate–adsorbate interactions

**Fig. 3:**
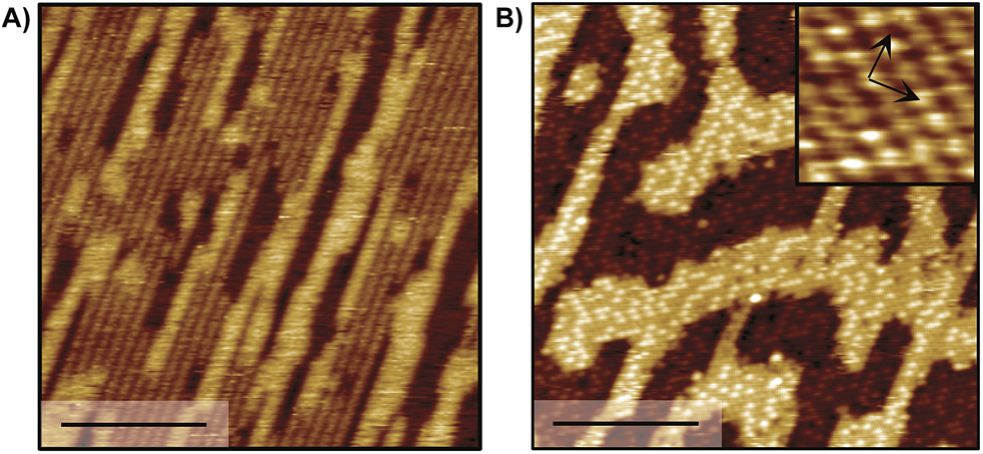
STM images of (A) 0.06 ML and (B) 0.19 ML trifluoroacetate on Au(110) demonstrate that carboxylates form dense local island at low global coverages; scale bar, 10 nm, sample bias: 0.5–1.5 V, tunneling current: 0.1 nA. Inset in B reveals the *c*(2 × 2) (∼400 K annealed surface in B).

Previous work demonstrated that acetate forms dense, self-assembled 2-D islands on Au(110) due to intermolecular interactions,^43^ suggesting that adsorbate–adsorbate interactions are likely to contribute to the stability of carboxylates in general. The adsorbate-induced “deconstruction” of the surface occurs even for extremely low acetate coverages, indicating a strong intermolecular attraction. In fact, STM experiments determined that the formation of dense *c*(2 × 2) islands occurs for trifluoroacetate and is accompanied by reconstruction of the gold surface from 1 × 2 to 1 × 1, similar to acetate (Fig. 3A, B). In particular, the same type of elongated feature is observed at low coverage (Fig. 3A) and a bimodal distribution of high and low domains at saturation coverage (Fig. 3B). Nucleation mechanisms associated with those features have been discussed previously in the case of acetate^43^ and are beyond the scope of this study. Likewise, the same molecular ordering is observed for formate and propanoate at saturation coverage (0.25 ML) based on LEED experiments (Fig. S9†), providing a guideline for further simulations including inter-adsorbate interactions. The bright protrusions observed for 0.19 ML trifluoroacetate on Au(110) are 39 ± 4 pm taller than the darker protrusions and are attributed to minor structural disorder that can be removed by mild annealing (Fig. S10†).

**Fig. 4:**
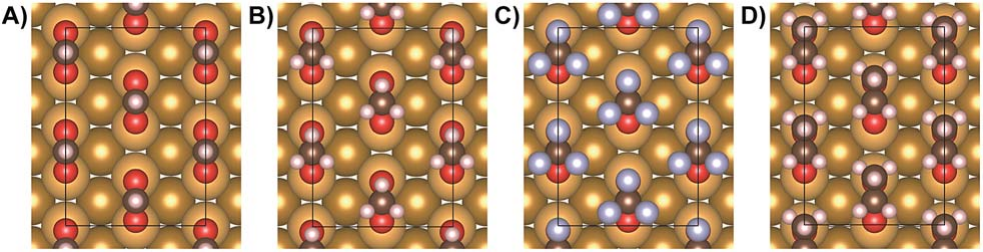
Geometries used in calculations modeling densely-packed carboxylates: (A) formate, (B) acetate, (C) trifluoroacetate, (D) propanoate. A *c*(2 × 2) unit cell was used for all cases, based on experimental measurements (STM and LEED).

The effect of adsorbate–adsorbate interactions on the stability of carboxylates on Au(110) is determined by comparing the calculated interface energy (*E*(interface)) of isolated carboxylates *versus* densely-packed species in a *c*(2 × 2) molecular arrangement (Fig. 4; Table 5; Fig. S11†). The method for calculating the interface energy is similar to the one used to describe acetate^43^ and is detailed in the ESI.†

**Table 5:** Interface energy change per adsorbate due to inter-adsorbate interaction (Δ*E*(interface)) and reaction energy as a condensed phase (Δ*E*(interface) + *E*(rxn)) calculated for each adsorbate, according to the method detailed in the ESI

	Δ*E*(interface) (eV)		Δ*E*(interface) + *E*(rxn) (eV)^*a*^	
	(PBE)	(PBE + vdW)	(PBE)	(PBE + vdW)
Propanoate	0.11	–0.10	–0.74	–1.14
Trifluoroacetate	0.17	–0.01	–0.80	–1.15
Acetate	0.11	–0.02	–0.71	–1.01
Formate	0.12	0.02	–0.77	–0.99

^*a*^ *E*(rxn) values are from Table 3.

In the absence of vdW interactions (PBE), the close packing associated with condensed islands is destabilizing for all carboxylates studied, as signified by the positive interface energy change, Δ*E*(interface) (Table 5; Fig. S11†). Furthermore, the interface energy change per adsorbate is essentially independent of the alkyl chain length, although the effect of the electron-rich CF_3_ group in trifluoroacetate is marginally more destabilizing. Although the energy difference per adsorbate is small, there are 4 adsorbates per supercell which makes the energy differences significant for condensed islands of carboxylates (Fig. 4).

Inclusion of vdW interactions for the condensed (2 × 2) layers leads to stabilization and improves the agreement between theory and experiment (Table 5). The overall stabilities (*E*(reaction) + Δ*E*(interface)) of formate and acetate are essentially the same and the propanoate is most strongly bound, in agreement with experiment (Table 2). The outlier is trifluoroacetate, which is predicted to have a similar overall stability as propanoate (Table 5; Fig. S11†), whereas in experiment propanoate binding is favored over trifluoroacetate (Table 2). Although the overall stability is similar for propanoate and trifluoroacetate, the underlying factors contributing to the stability are different. The primary binding of the carboxylate functionality to the surface is stronger for trifluoroacetate, 1.14 eV; whereas the primary binding of the propanoate is lower, 1.05 eV. On the other hand, the propanoate gains more stability from the inter-adsorbate interaction, Δ*E*(interface) = 0.10 eV. Hence, the overall binding is a combination of different effects. It is possible, even likely, that errors in the calculated energies for these two effects combine to yield a similar overall stability even though these two carboxylates have different binding efficacies.

Although the DFT calculations are fairly accurate for comparing the stability of carboxylate because of their similar adsorption geometry, errors in the relative binding energies could still potentially be as large as several tens of meV. Based on the equilibrium constant measurements, the difference in free energy between the most stable carboxylate, propanoate, and the least stable carboxylate, formate, is 0.04 eV. Hence, the calculations and the results, are in general agreement with the experimental measurements. Nevertheless, the accuracy of the DFT calculations is not sufficient to quantitatively predict the energetic differences. Further, the DFT calculations do not take into account the role of entropy in the displacement experiments which could have a minor but possibly significant contribution in determining the displacement trends. We therefore focus on the qualitative effects of the vdW interaction and inter-adsorbate interaction on the stability of each adsorbate.

The overall hierarchy of stability for carboxylates depends on their condensation into islands, rendering longer-chain carboxylates more stable as shown for assembly of alkanethiols on Au(111) for carbon chains up to ten carbons.^53^ Accordingly, adsorbate–adsorbate stabilization calculated for trifluoroacetate is the same as for acetate, consistent with a driving force for trifluoroacetate to form dense *c*(2 × 2) molecular domains at low coverage and in agreement with the STM results that are similar to those for acetate. The attraction occurs despite the higher electron density that could lead to larger repulsive coulombic interactions.

The effect of carboxylate islanding is not unique to gold^43^,^54^ but rather has been shown to occur generally on metal surfaces, including: Cu,^55^–^59^ Ag,^60^ Al,^60^ Ni^61^,^62^ and Pd.^63^,^64^ Therefore, we anticipate that the effect of intermolecular interactions on carboxylate stability demonstrated herein would be a necessary consideration to carboxylate-metal systems.

## Conclusions

Experimental work determined that carboxylate species, while strongly bound to the surface, have a much weaker dependence of stability on alkyl chain length compared to alkoxides. DFT calculations suggest that the rigid bidentate structure limits the influence of adsorbate-surface vdW interactions on carboxylate stability, accounting for this weak dependence. A second important factor in determining the stability of carboxylates are adsorbate–adsorbate interactions that drive condensation of the carboxylates into condensed islands on Au(110). Even for chains as short as acetate and trifluoroacetate, these interactions are sufficiently strong to drive the “deconstruction” of the Au(110) surface so the condensed (2 × 2) structure forms. The effect of adsorbate–adsorbate interactions on carboxylate stability demonstrated here is likely universal for bidentate carboxylate binding on metallic substrates due to the ubiquitous observation of carboxylate islanding on metal surfaces. The anchoring geometry controls the relative influence of adsorbate-surface and adsorbate–adsorbate vdW interactions which independently can control intermediate stability. These studies further demonstrate that complexity, such as formation of condensed phases, must be taken into account in building up an understanding of the binding of key intermediates and poisons on surfaces. Hence, a combination of experimental measurement and theoretical calculations are required to develop a complete picture.

## Conflicts of interest

There are no conflicts to declare.

## Supplementary Material

Supplementary information
