# Supplementary material for: Identifying key descriptors in surface binding: interplay of surface anchoring and intermolecular interactions for carboxylates on Au(110)
Source: Chem Sci. 2018 Mar 12;9(15):3759–66. doi: 10.1039/c7sc05313d (PMC5939607; doi:10.1039/c7sc05313d)
Supplement: Supplementary file 1 [file SC-009-C7SC05313D-s001.pdf]

Electronic Supplementary Material (ESI) for Chemical Science.
This journal is © The Royal Society of Chemistry 2018

Supplemental Information

Identifying key descriptors in surface binding: Interplay of surface anchoring and intermolecular interactions for carboxylates on Au(110)

Christopher R. O'Connor,[†a] Fanny Hiebel,[†a] Wei Chen,[b,c] Efthimios Kaxiras,[b,c] Robert J. Madix[b] and Cynthia M. Friend[*a,b]

[a] Department of Chemistry and Chemical Biology, Harvard University, Cambridge, MA 02138, USA.

[b] School of Engineering and Applied Sciences, Harvard University, Cambridge, MA 02138, USA.

[c] Department of Physics, Harvard University, Cambridge, MA 02138, USA.

Detailed experimental methods for temperature programmed reactions

Temperature programmed experiments were performed with each reactant to identify its signature products. Each experiment surveyed all masses in the range of m/z = 2 to m/z = 2m, where m is the molecular mass of the heaviest compound, to ensure that all reactants and products were detected. First, a monolayer of RCOOH was adsorbed to the O/Au(110) surface at 130 K, and the temperature at which RCOOH could be dosed to form isolated $RCOO_{ads}$ was determined by noting the temperature at which resultant water and excess RCOOH were desorbed. A monolayer of RCOOH was then dosed at this predetermined temperature to completely react adsorbed O and desorb the product H_2O. The isolated $RCOO_{(a)}$ was cooled to 130 K, TPRS was performed, and the characteristic decomposition products were detected.

Displacement reactions were performed to determine the relative binding stability of selected pairs of carboxylates. First isolated $RCOO_{(a)}$ was formed as previously described; then the isolated $RCOO_{(a)}$ was exposed to a monolayer of R'COOH at the predetermined temperature that would isolate either $RCOO_{(a)}$ or $R'COO_{(a)}$. Quantitative TPRS was then employed to determine the relative adsorbed amounts of $RCOO_{(a)}$ and $R'COO_{(a)}$ on the basis of the amounts of their characteristic decomposition products, appropriately corrected for their relative detection efficiencies and the selectivity of the decomposition reaction product as determined previously.

Procedure for determining the equilibrium constant of reversible displacement reactions in the limit of low coverage from temperature programmed reaction data

In the quantitative analysis, the consideration of the molecular fragmentation pattern, ionization cross-section, and the mass spectrometer transmission and detection coefficients is necessary. It has been shown elsewhere[1] that the number density of molecule i in the ionizer, n_i , is given as follows:

$$n_i = \frac{\sigma_i^{-1} \cdot s_{ik}}{T_{ik} \cdot \delta_{ik}} \cdot \left(1 + \sum_j \frac{s_{ij}}{s_{ik}} \cdot \frac{T_{ik} \cdot \delta_{ik}}{T_{ij} \cdot \delta_{ij}}\right) \qquad \text{(Eqn. S1)}$$

where

σ_i is the total ionization cross-section of molecule i,

s_{ik} is the measured signal current for the kth fragment of molecule i,

s_{ij}/s_{ik} is the ratio of signals of the jth and kth fragments of molecule i determined from separate calibrations of the neat parent molecule i,

T_{ik} is the transmission coefficient of a fragment with m/z, k, of molecule i,

T_{ij} is the transmission coefficient of a fragment with m/z, j, of molecule i,

δ_{ik} is the detection coefficient of a fragment with m/z, k, of molecule i,

δ_{ij} is the detection coefficient of a fragment with m/z, j, of molecule i.

In the equation above, the constants σ_i, T_{ik}, T_{ij}, δ_{ik} and δ_{ij} are taken from published values (Table S1). s_{ij}/s_{ik} is determined for molecule i by condensing a neat sample of molecule i on clean Au(110) and the fragmentation pattern was recorded by TPRS.

Table S1. Mass Spectrometry quantitative analysis constants

**m/z**	**Transmission Coefficient** [a]	**Detection Coefficient** [b]	**Molecule**	**Ionization Cross-section**
0-20	1	1.5	CO_2	3.521[c]
21-30	1	1	CH_3	3.098[c]
31-40	1	0.9	CF_3	4.540[c]
41-50	1	0.8	CH_2CH_2	5.115[c]
51-60	1	0.7	HCOOH	5.09[d]
61-70	1	0.65		
71-80	0.98	0.6		

[a] Adapted from Hiden manual
[b] Adapted from UTI manual
[c] Values calculated at an incident electron voltage of 70 eV from the NIST database
[d] Adapted from reference 2

The equilibrium constant of the displacement reaction (Eqn. S2) can be determined from the forward and reverse direction displacement experiments:

$$RCOO_{(a)} + R^{'}COOH_{(g)} \leftrightarrow RCOOH_{(g)} + R^{'}COO_{(a)} \qquad \text{(Eqn. S2)}$$

Experimentally, the surface concentration of the two carboxylates could be quantitatively determined by the generalized formula (Eqn. S3). The selectivity fraction, i.e. the fraction of carboxylate that forms product molecule i, for each adsorbate was determined by independent oxidation experiments.

$$\theta_R = f_i \cdot n_i \qquad \text{(Eqn. S3)}$$

where

θ_R is the coverage of RCOO prior to decomposition,

n_i is the amount of product molecule i produced from the decomposition of RCOO,

f_i is the selectivity fraction of RCOO that decomposes to product molecule i.

Under the reaction conditions, the desorption temperature of the carboxylic acids (RCOOH, R′COOH) is much lower than the reaction temperature and the rapid pumping of the gas phase species away from the surface gives the rate of displacement as:

$$Rate = -\frac{d\theta_R}{dt} = A_f \cdot J_{R^{'}H} \cdot \theta_R \cdot \exp\left[\frac{-E_f}{R \cdot T}\right] \qquad \text{(Eqn. S4)}$$

where

A_f is the pre-exponential for the forward reaction (ML^{-1}),

$J_{R^{'}H}$ is the molecular flux of $R^{'}COOH_{(g)}$ (ML/s),

θ_R is the coverage of $RCOO_{(a)}$ (ML),

E_f is the activation energy of the forward displacement reaction (J/mol),

R is the gas constant (J/mol K),

T is the temperature of the sample.

We know that at $t = 0\ s$, $\theta_R = \theta_R^i$ where θ_R^i is the initial coverage of RCOO and at $t = t_f$, $\theta_R = \theta_R^f$ where θ_R^f is the final coverage of RCOO. The initial coverage of RCOO can be determined from the total amount of all products desorbed and is confirmed by a calibration dose of oxygen recombination. The final coverage of RCOO can be determined from the amount of product from RCOO detected. Solving the differential equation gives Eqn. S5.

$$-\int_{\theta_R^i}^{\theta_R^f} \frac{d\theta_R}{\theta_R} = \int_0^{t_f} A_f \cdot J_{R'H} \cdot \exp\left[\frac{-E_f}{R \cdot T}\right] \cdot dt$$

$$-\ln\left(\theta_R^f\right) + \ln\left(\theta_R^i\right) = A_f \cdot J_{R'H} \cdot \exp\left[\frac{-E_f}{R \cdot T}\right] \cdot t_f - 0$$

$$-\ln\left(\frac{\theta_R^f}{\theta_R^i}\right) = A_f \cdot J_{R'H} \cdot \exp\left[\frac{-E_f}{R \cdot T}\right] \cdot t_f$$

$$\ln\left(1 + \frac{\Delta\theta_R}{\theta_R^i}\right) = -A_f \cdot J_{R'H} \cdot t_f \cdot \exp\left[\frac{-E_f}{R \cdot T}\right]$$

$$\frac{\ln\left(1 + \frac{\Delta\theta_R}{\theta_R^i}\right)}{-A_f \cdot J_{R'H} \cdot t_f} = \exp\left[\frac{-E_f}{R \cdot T}\right]$$

$$\ln\left(\frac{\ln\left(1 + \frac{\Delta\theta_R}{\theta_R^i}\right)}{-A_f \cdot J_{R'H} \cdot t_f}\right) = \frac{-E_f}{R \cdot T}$$

$$E_f = -R \cdot T \cdot \ln\left(\ln\left(1 + \frac{\Delta\theta_R}{\theta_R^i}\right) \Big/ \left(-A_f \cdot J_{R'H} \cdot t_f\right)\right) \qquad \text{(Eqn. S5)}$$

where

$\Delta\theta_R$ is change in $RCOO$ coverage by the displacement process (ML),

$\theta_{R'}^{i}$ is the initial coverage of $RCOO_{(a)}$ (ML),

t_f is the dosing time of molecule $R'COOH_{(g)}$ (s).

Likewise the displacement reaction was probed from the reverse direction and the same derivation produces the following:

$$E_r = -R \cdot T \cdot \ln\left(\ln\left(1 + \frac{\Delta\theta_{R'}}{\theta_{R'}^i}\right) \Big/ \left(-A_r \cdot J_{RH} \cdot t_f'\right)\right) \qquad \text{(Eqn. S6)}$$

where

E_r is the activation energy of the reverse displacement reaction (J/mol),

$\Delta\theta_{R'}$ is the change in $R'COO$ coverage by the displacement process (ML),

$\theta^{i}_{R'}$ is the initial coverage of $R'COO_{(a)}$ (ML),

A_r is the pre-exponential for the reverse reaction (ML^{-1}),

J_{RH} is the molecular flux of $RCOOH_{(g)}$ (ML/s),

t'_f is the dosing time of molecule $RCOOH_{(g)}$ (s).

The equilibrium constant can be derived from E_f and E_r (Eqn. S7) and thus evaluated from the forward and reverse direction displacement experiments. For clarity, square brackets are used in Eqn. S7 to separate the information measured by each experiment.

$$\Delta E = E_f - E_r$$

$$\Delta E = -R \cdot T \cdot \ln\left(\frac{\ln\left(1 + \frac{\Delta\theta_R}{\theta^i_R}\right)}{A_f \cdot J_{R'H} \cdot t_f} \cdot \frac{A_r \cdot J_{RH} \cdot t'_f}{\ln\left(1 + \frac{\Delta\theta_{R'}}{\theta^i_{R'}}\right)}\right)$$

$$K = \exp\left(-\frac{\Delta E}{RT}\right)$$

$$K = \left[\ln\left(1 + \frac{\Delta\theta_R}{\theta^i_R}\right) \Big/ \left(A_f \cdot J_{R'H} \cdot t_f\right)\right] \cdot \left[\left(A_r \cdot J_{RH} \cdot t'_f\right) \Big/ \ln\left(1 + \frac{\Delta\theta_{R'}}{\theta^i_{R'}}\right)\right] \quad \text{(Eqn. S7)}$$

Assuming that the pre-exponential factor for the forward and reverse reaction is the same, $\frac{A_r}{A_f} \cong 1$, this simplifies to:

$$K = \left[\ln\left(1 + \frac{\Delta\theta_R}{\theta^i_R}\right) \Big/ \left(J_{R'H} \cdot t_f\right)\right] \cdot \left[\left(J_{RH} \cdot t'_f\right) \Big/ \ln\left(1 + \frac{\Delta\theta_{R'}}{\theta^i_{R'}}\right)\right] \quad \text{(Eqn. S8)}$$

For temperatures above the decomposition temperature of an adsorbate the molecular flux cannot be measured directly and needs to be appropriately calibrated by a condensation experiment. At temperatures much lower than the desorption temperature, the adsorption rate of a physisorbed molecule is given as follows:

$$\frac{d\theta_{R'H}}{dt} = S \cdot J_{R'H} \quad \text{(Eqn. S9)}$$

where S is the sticking probability.

The molecular flux can be determined by measuring the amount of molecules desorbed from a fixed molecular exposure with a time length t_f (Eqn. S10)

$$\int_0^{\theta^f_{R'H}} d\theta_{R'H} = \int_0^{t_f} S \cdot J_{R'H} \cdot dt$$

$$\Delta\theta_{R'H} = S \cdot J_{R'H} \cdot t_f \qquad \text{(Eqn. S10)}$$

This can be simplified by assuming the sticking probability for the condensation is unity, $S \cong 1$ (Eqn. S11).

$$\Delta\theta_{R'H} = J_{R'H} \cdot t_f \qquad \text{(Eqn. S11)}$$

The equilibrium constant can now be determined using the measureable quantities from the displacement (Forward: $\Delta\theta_R$, θ^i_R , Reverse: $\Delta\theta_{R'}$, $\theta^i_{R'}$) and calibration ($\Delta\theta_{R'H}$, $\Delta\theta_{RH}$) experiments (Eqn. S12).

$$K = \left[\ln\left(1 + \frac{\Delta\theta_R}{\theta^i_R}\right) \Big/ \Delta\theta_{R'H} \right] \cdot \left[\Delta\theta_{RH} \Big/ \ln\left(1 + \frac{\Delta\theta_{R'}}{\theta^i_{R'}}\right) \right] \qquad \text{(Eqn. S12)}$$

Experimental peak deconvolution for Figure 1

The deconvoluted CO_2 signal for acetate is calculated by

$$S_{acetate}(CO_2) = S(CH_3) \cdot \frac{I(CO_2)}{I(CH_3)} \qquad \text{(Eqn. S13)}$$

where $S_{acetate}(CO_2)$ is the deconvoluted acetate signal, $S(CH_3)$ is the measure CH_3 signal, and $\frac{I(CO_2)}{I(CH_3)}$ is the selectivity ratio of CO_2 to CH_3 measured for isolated acetate.

The deconvoluted CO_2 signal for propanoate is calculated by

$$S_{propanoate}(CO_2) = S(CF_3) \cdot \frac{I(CO_2)}{I(CF_3)} \qquad \text{(Eqn. S14)}$$

where $S_{propanoate}(CO_2)$is the deconvoluted propanoate signal, $S(CF_3)$is the measure CF_3 signal, and $\frac{I(CO_2)}{I(CF_3)}$ is the selectivity ratio of CO_2 to CF_3 measured for isolated trifluoroacetate.

The sum of the deconvoluted CO_2 signal calculated for acetate and trifluoroacetate is in excellent agreement with the measured CO_2 signal which confirms that the product selectivity is coverage independent below 0.10 ML of acetate and trifluoroacetate.

Experimental results for the stability hierarchy in Table 2.

Formic acid + Acetic acid

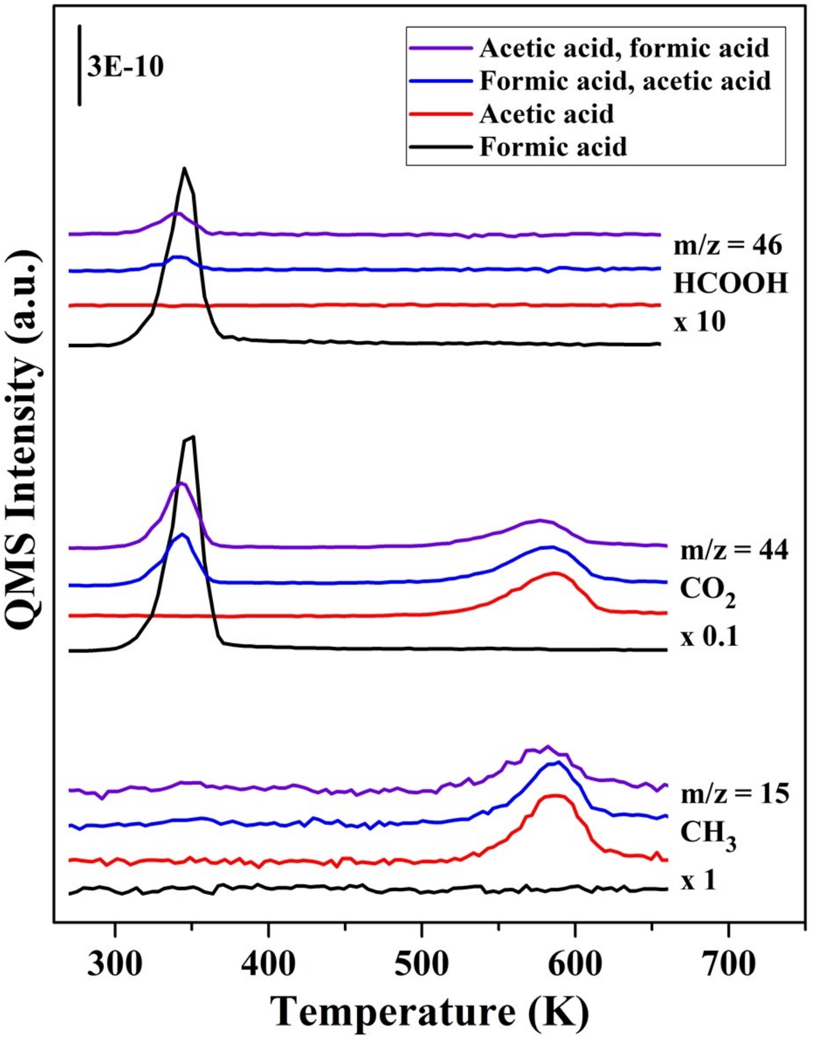

Figure S1: In separate experiments, ~1 ML of formic acid (black) and acetic acid (red) were dosed on 0.05 ML O/Au(110) at 260 K to establish their reactivity. Then acetic acid was introduced on isolated formate/Au(110) at 260 K (blue), and formic acid was introduced on isolated acetate/Au(110) at 260 K (purple).

In the competitive displacement experiments between formic acid and acetic acid, the product distribution is indicative of a majority amount of acetate on the surface, as evidenced by CO_2 and CH_3 at 580 K. The product distribution indicates that there is a minority amount of formate on the surface, as evidenced by CO_2 and HCOOH at 350 K. The displacement of formate by acetic acid and acetate by formic acid yield a similar product distribution which indicates that the two carboxylate species exist in a reversible equilibrium that favors acetate.

Acetic acid + Propanoic acid

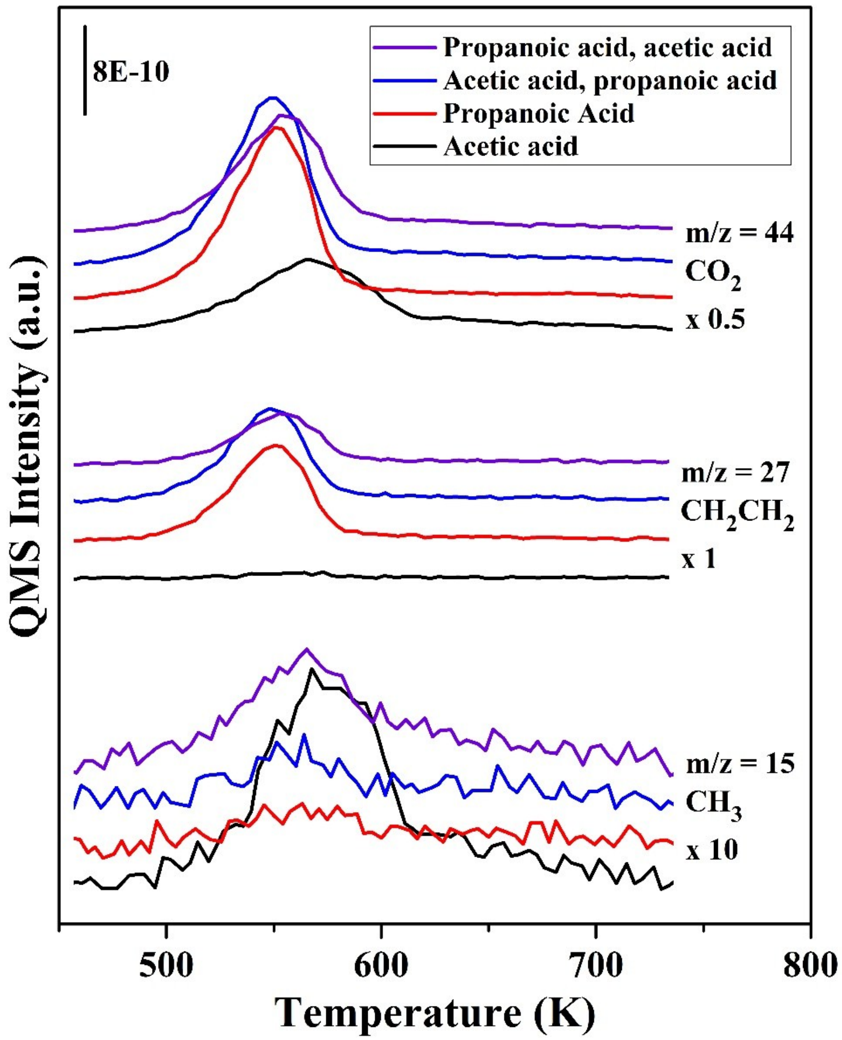

Figure S2: In separate experiments, ~1 ML of acetic acid (black) and propanoic acid (red) were dosed on 0.05 ML O/Au(110) at 300 K to establish their reactivity. Then propanoic acid was introduced on isolated acetate/Au(110) at 300 K (blue), and acetic acid was introduced on isolated propanoate/Au(110) at 300 K (purple).

In the competitive displacement experiments between acetic acid and propanoic acid, the product distribution is indicative of a majority amount of propanoate on the surface, as evidenced by CO_2 and CH_2CH_2 at 550 K. The product distribution indicates that there is a minority amount of acetate on the surface, as evidenced by CO_2 and CH_3 at 580 K. The displacement of acetate by propanoic acid and propanoate by acetic acid yield a similar product distribution which indicates that the two carboxylate species exist in a reversible equilibrium that favors propanoate.

Trifluoroacetic acid + Propanoic acid

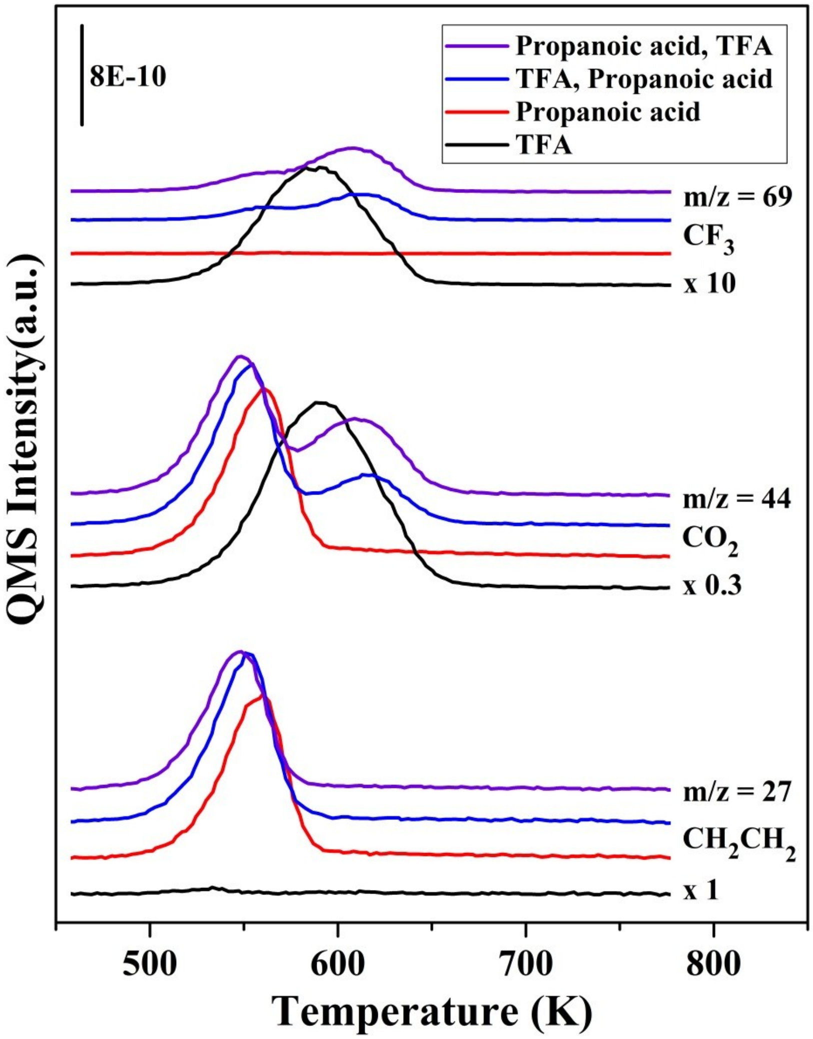

Figure S3: In separate experiments, ~1 ML of trifluoroacetic acid (black) and propanoic acid (red) were dosed on 0.05 ML O/Au(110) at 300 K to establish their reactivity. Then propanoic acid was introduced on isolated trifluoroacetate/Au(110) at 300 K (blue), and trifluoroacetic acid was introduced on isolated propanoate/Au(110) at 300 K (purple).

In the competitive displacement experiments between trifluoroacetic acid and propanoic acid, the product distribution is indicative of a majority amount of propanoate on the surface, as evidenced by CO_2 and CH_2CH_2 at 550 K. The product distribution indicates that there is a minority amount of trifluoroacetate on the surface, as evidenced by CO_2 and CF_3 at 590 K. The displacements of trifluoroacetate by propanoic acid and propanoate by acetic acid yield a similar product distribution which indicates that the two carboxylate species exist in a reversible equilibrium that favors propanoate.

Equilibrium constant determination for acetic acid and propanoic acid

$$K = \left[\ln\left(1 + \frac{\Delta\theta_{CH_3COO}}{\theta^{i}_{CH_3COO}}\right) \Big/ \Delta\theta_{CH_3CH_2COOH} \right] \cdot \left[\Delta\theta_{CH_3COOH} \Big/ \ln\left(1 + \frac{\Delta\theta_{CH_3CH_2COO}}{\theta^{i}_{CH_3CH_2COO}}\right) \right] \quad \text{(Eqn. S12)}$$

The reaction pair of acetic acid and propanoic acid demonstrates the calculation of the equilibrium constant for a displacement experiment. The exposures of propanoic acid ($\Delta\theta_{CH_3CH_2COOH}$) and acetic acid ($\Delta\theta_{CH_3COOH}$) used in the displacement experiments were calibrated by condensation of the respective organic acid to the clean gold surface at 130 K. The initial coverage of acetate ($\theta^{i}_{CH_3COO}$) was determined by quantitative analysis of pure acetate formed from a calibrated precoverage of O. The change in coverage of acetate ($\Delta\theta_{CH_3COO}$) by the calibrated exposure of propanoic acid ($\Delta\theta_{CH_3CH_2COOH}$) was determined by quantitative analysis of the signature products of acetate compared to the pure acetate formed from a calibrated precoverage of adsorbed O. The initial coverage of propanoate ($\theta^{i}_{CH_3CH_2COO}$) was determined by quantitative analysis of pure propanoate formed from a calibrated precoverage of O. The change in coverage of propanoate ($\Delta\theta_{CH_3CH_2COO}$) due to the calibrated exposure of acetic acid ($\Delta\theta_{CH_3COOH}$) was determined by quantitative analysis of the signature products of propanoate compared to a known coverage of pure propanoate. These calculated values assume that the selectivity of the product signatures for the decomposition of acetate and propanoate when mixed do not differ from the pure species. The equilibrium constant between propanoate and acetate is 4; propanoate is more stable than acetate (Figs. S2, S4). The same methodology was employed to calculate the equilibrium constants for displacement experiments of other acid pairs.

Kinetic model for displacement reactions predictions

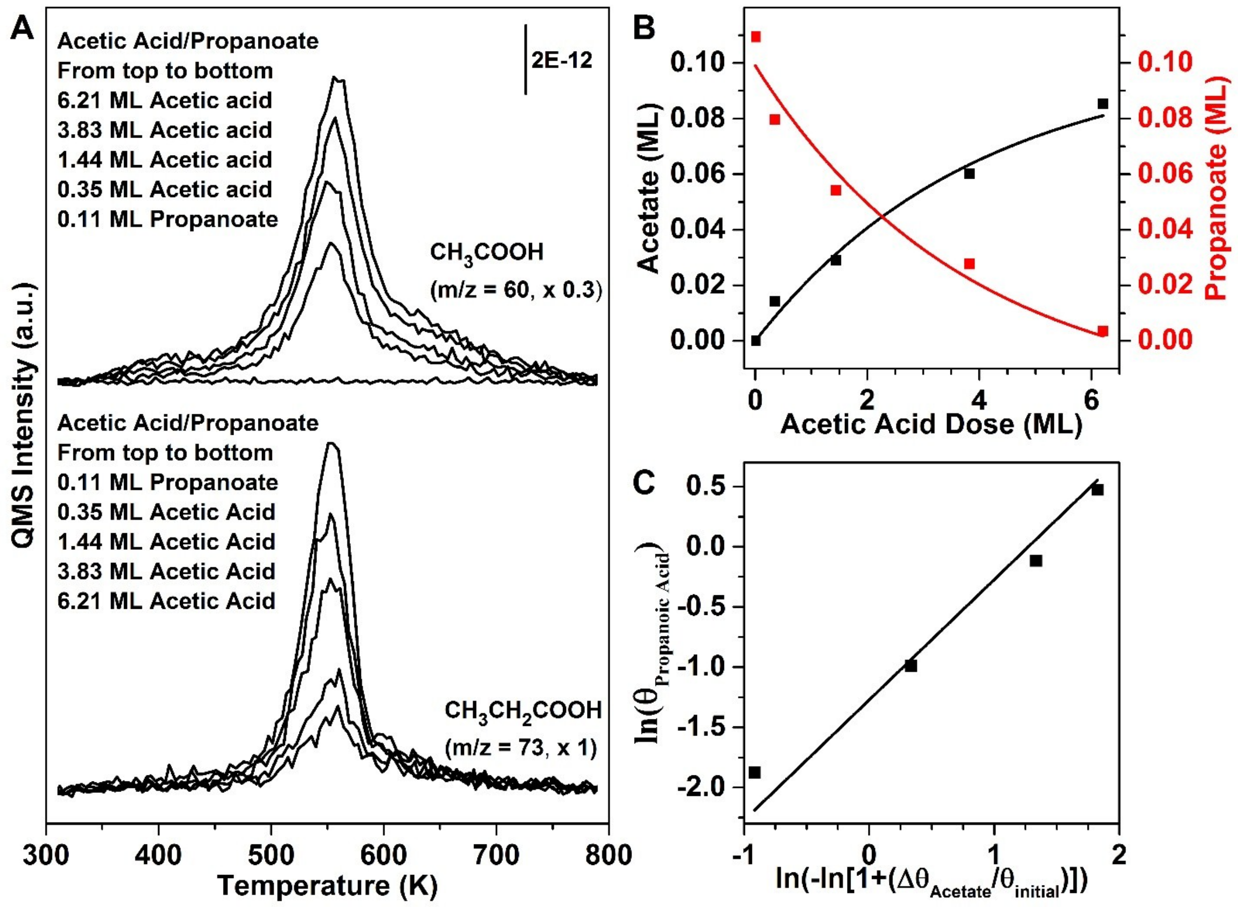

Figure S4. The extent of displacement of propanoate by acetate monotonically increases with the amount of acetic acid dosed. (A) Temperature programmed experiments are used to measure the extent of displacement of propanoate by acetate which can be controlled by the exposure of acetic acid (0.35 ML, 1.44 ML, 3.83 ML, 6.21 ML) to a 0.11 ML propanoate covered gold surface. (B) The surface concentration of propanoate (red data point) and acetate (black data point) for a specified dose of acetic acid as predicted using Eqn. S15 (red curve) and 11 (black curve) respectively. (C) A curve fit of the data according to Eqn. S16 (R^2 =0.956) demonstrates that the pre-factor and activation energy can be treated as constant for the displacement over the entire range of propionate coverage.

The validity of this approach used to determine K was demonstrated by the prediction of the relative surface concentration of acetate and propanoate resulting from a well-defined increase in the acetic acid exposure to adsorbed propanoate (Figure S4). The concentration of acetate or propanoate was determined from their signature products acetic acid, m/z = 60 and 73, respectively (Figure S4A, S4B). The surface concentration of acetate or propionate are given by Eqns. S15 and S16, respectively

$$\theta_{CH_3CH_2COO} = \theta^{i}_{CH_3CH_2COO} \cdot \exp\left[-A \cdot \Delta\theta_{CH_3COOH} \cdot e^{-\frac{E}{R \cdot T}} \right] \qquad \text{(Eqn. S15)}$$

$$\theta_{CH_3COO} = \theta^{i}_{CH_3CH_2COO} \cdot \exp\left[-A \cdot \Delta\theta_{CH_3COOH} \cdot e^{-\frac{E}{R \cdot T}} \right] - \theta^{i}_{CH_3CH_2COO} \qquad \text{(Eqn. S16)}$$

where A is the pre-exponential factor (ML^{-1}) and E is the activation energy of the displacement reaction. There is good agreement between the experimentally determined surface concentration of acetate and propanoate and the model curve fit ($R^2 = 0.986$ and 0.950, respectively) (Figure S4B).

The kinetic parameters of the displacement of propanoate by acetate are essentially coverage independent. By rearranging Eqn. S15, the dependence of the change in surface concentration on the activation energy and pre-exponential factor for displacement gives (Eqn. S17)

$$\ln\left(\Delta\theta_{CH_3COOH}\right) = \ln\left(-\ln\left[1 + \frac{\Delta\theta_{CH_3CH_2COO}}{\theta^{i}_{CH_3CH_2COO}} \right] \right) - \ln(A) + \frac{E}{R \cdot T} \qquad \text{(Eqn. S17)}$$

The data fits this relationship well ($R^2 = 0.956$) (Figure S4C); this indicates that the pre-exponential factor and activation energy are essentially coverage independent over the coverage range studied.

Numerical simulations

Initial adsorption geometry explorations were performed to discriminate between monodentate top, bidentate bridge, bidentate top and chelating geometries for formate and propanoate (Figs. S5 and S6 resp.). Clearly, the preferred adsorption geometry is bidentate top for all carboxylates, similarly to what has been reported for acetate.[4] Other geometries are either unstable and therefore relax into bidentate top (arrows) or lead to considerably higher total energy, as indicated in the figures.

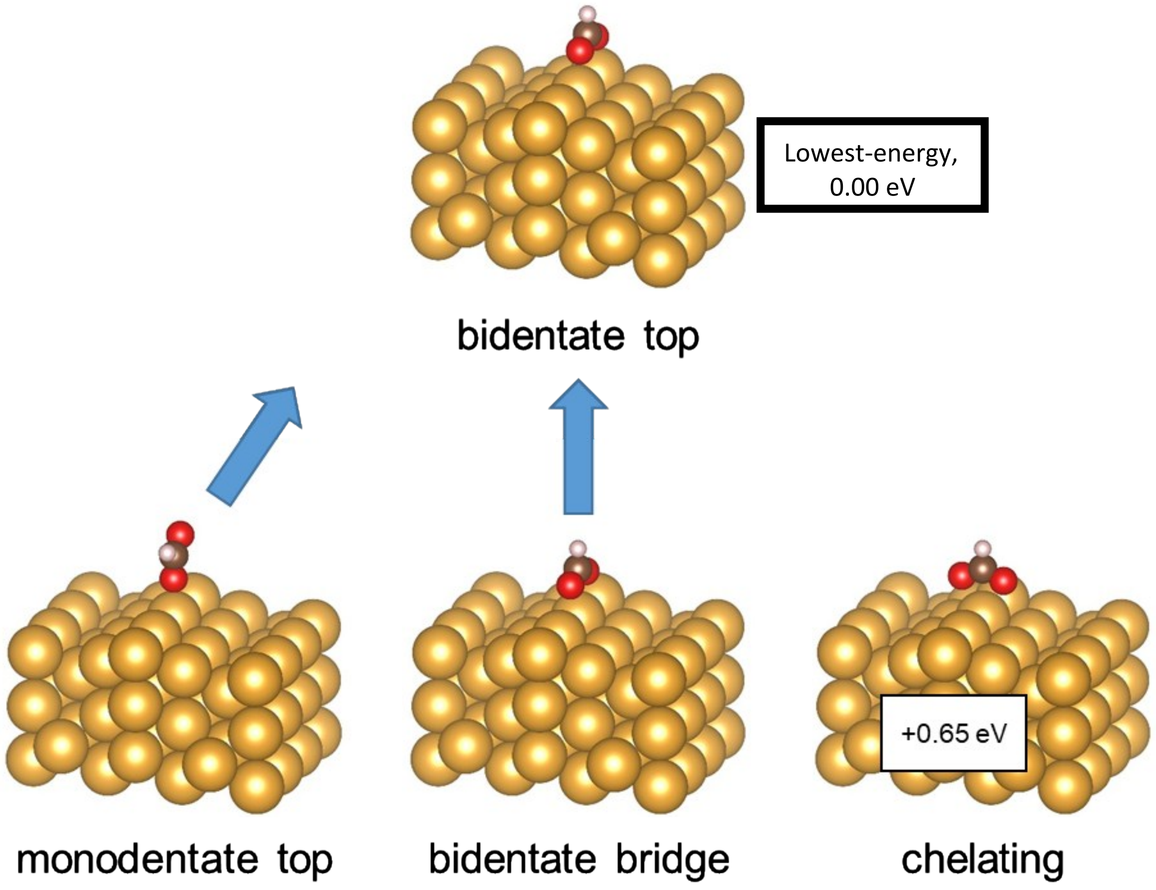

Figure S5: Exploration of the adsorption geometry of formate. The most stable geometry is bidentate top, chelating is +0.65 eV less stable and other configurations relax into bidentate top.

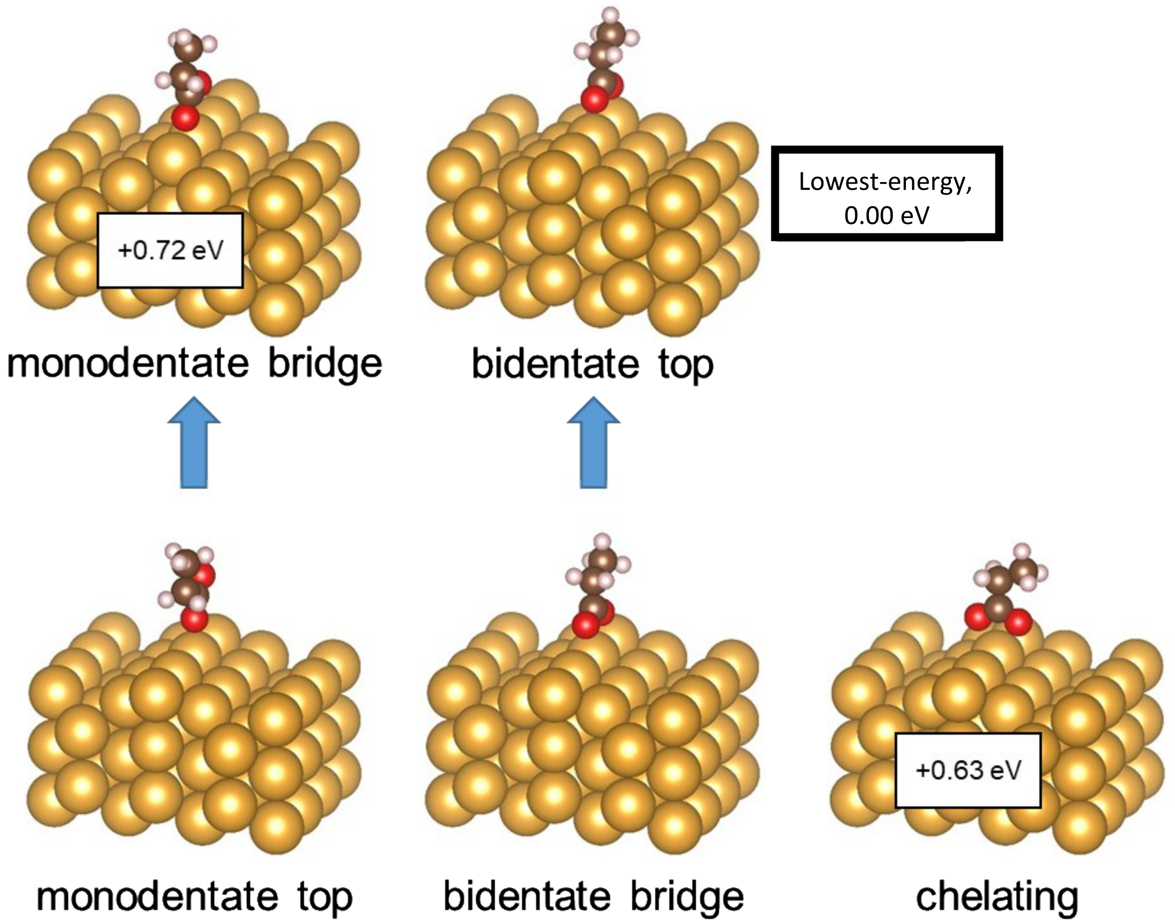

Figure S6: Exploration of the adsorption geometry of propanoate. The most stable geometry is bidentate top, chelating is +0.63 eV less stable, monodentate top relaxes into monodentate bridge which is +0.73 eV less stable and bidentate bridge relaxes into bidentate top.

Through a combination of strong corrugation of the surface and molecule-surface vdW interactions, a quasi-degeneracy in adsorption site emerges for isolated propanoate. The effect is significantly smaller for acetate. PBE_{SP} calculations in the configuration given in Fig. S7 yield similar energy loss compared to the top-layer adsorption, amounting to 0.41 eV. However, the total energy i.e. including vdW contributions, is 0.12 eV higher compared to vertical for acetate. For propanoate the difference is 3 times smaller (0.04 eV). A vdW contribution of 0.37 eV is extracted for propanoate, which is close enough to compensate for the loss of energy due to sub-optimal binding to Au. Acetate exhibits a lower stabilization of 0.29 eV, because it has one fewer methyl group interacting with the surface. In both cases, the molecule-Au surface distances are consistent with a stronger vdW surface-molecule interaction than in its top-layer configuration (Table S2).

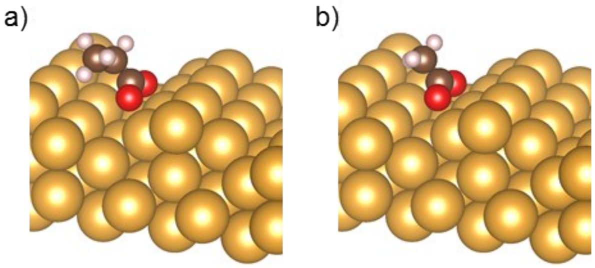

Figure S7: Relaxed propanoate (a) and acetate (b) geometries bonded on the second Au layer from the top, in bidentate top configuration. Acetate (propanoate) is 0.12 eV (0.04 eV) less stable than the top-row configuration.

	propanoate	acetate
h_{C0} (Å)	3.3	3.4
h_{C1} (Å)	3.8	3.9
h_{C2} (Å)	4.0	

Table S2: Distance between the C atom and the (111) microfacet in the configuration in Fig. S7.

Molecular tilting and rotation for isolated propanoate

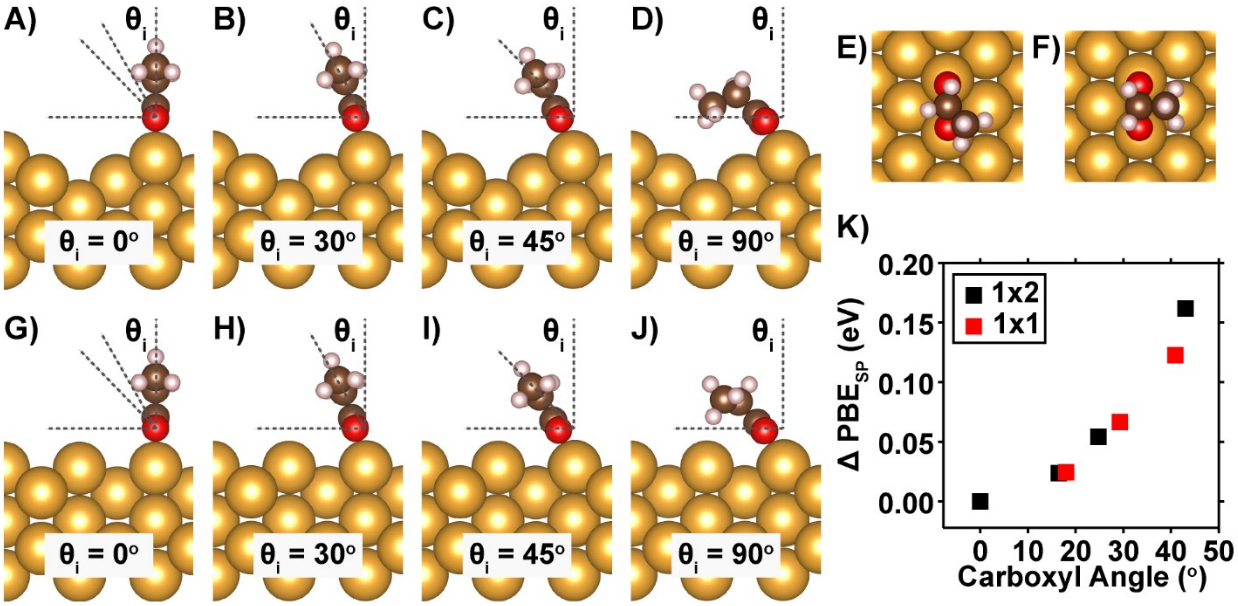

Figure S8: Relaxed configurations for propanoate on (1×2) (A-F) and on (1×1) (G-J) from initial geometry tilted with respect to the OO axis by 0-90˚ (A-D and G-J) and rotated with respect to CC axis by 45-90˚ (E-F). The respective weight of vdW and PBE contributions is represented in (K) by comparing van der Waals corrected (vdW) and PBE electronic relaxation (PBE_{SP}) as a function of the angle between the surface normal and the carboxyl group in the relaxed structure.

The impact of tilting the molecular plane towards the surface—with respect to the surface normal—and internal rotation - with respect to the C_0C_1 bond- were investigated for isolated carboxylate molecules (Fig. S8). The tilted configurations are never more stable than the upright configuration. In fact, energy differences are small, below ~25 meV or k_BT at 300K, in all relaxed configurations, and this observation is valid for both (1×2) and (1×1). The small energy difference over a wide range of angles indicates that the alkyl chain of an isolated molecule is highly mobile around its anchoring point at room temperature which contributes to the configurational entropy of adsorption. In contrast, internal rotation does not play an important role in the stability of the molecule on the surface.

Ionic relaxation of structures where the molecular plane has been tilted with respect to the normal surface always tends to bring the carboxyl group back into alignment with the Au center; this demonstrates the directional nature of the Au-O bond, likely because of an electronic resonance phenomenon. By comparing vdW ionic and electronic relaxed structures to PBE-only electronic relaxation (PBE_{SP}), as the angle of tilting increases the PBE contribution is reduced ($\Delta PBE_{SP}>0$) but is compensated by an increase in vdW interactions with the surface which indicates that the directional molecular orientation restricts strong interactions of the alkyl groups with the surface (Fig. S8K). For the same initial rotation angle, the relaxed molecule is systematically closer to the surface on the unreconstructed 1×1 surface which necessitates large angles for the molecule to feel the presence of the 1×2 surface.

The stability of the molecular rotations and tilting shows that the stronger binding of carboxylates compared to alkoxides minimizes the role of adsorbate-surface vdW interactions for isolated carboxylates. In contrast with alkoxides, the carboxyl group does not allow the carbon chain to freely tilt toward the surface; alkoxide leaning is responsible for stronger vdW interactions with the substrate and eventually a strong chain-length dependent surface stability.

Low Energy Electron Diffraction (LEED) results for adsorbate ordering

LEED performed on clean Au(110) confirms the (1×2) "missing row" reconstruction of the surface (Figs. S9C, S9E, S9G).[3] It has been previously shown using scanning tunneling microscopy (STM) and theoretical calculations that acetate induces a reconstruction of the Au-(1×2) to Au-(1×1) and forms a c(2×2) adsorbate ordering.[4] LEED experiments demonstrate that the characteristic LEED pattern observed for acetate (Fig. S9D), which has a c(2×2) adsorbate ordering based on STM experiments, occurs as well as for formate (Fig. S9F) and propanoate (Fig. S9H). The c(2×2) reconstruction is only visible for several seconds before beam damage affects the molecular ordering and leaves the Au-(1×1) visible. A theoretical p(1x2) and c(2x2) LEED pattern schematic is shown for reference (Figure S9A-B).

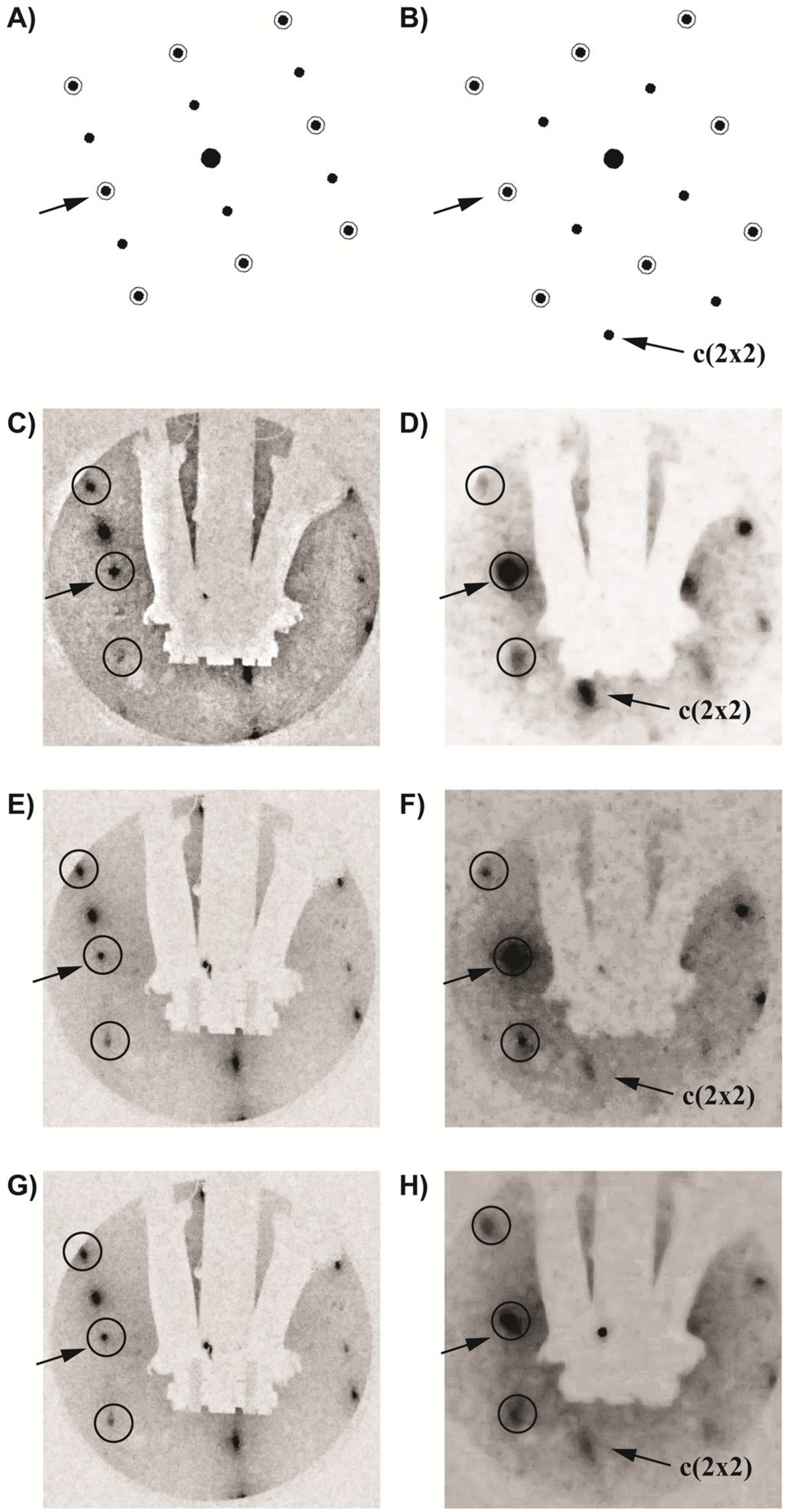
A)
B)
c(2x2)
C)
D)
c(2x2)
E)
F)
c(2x2)
G)
H)
c(2x2)

Figure S9: A) LEED pattern for an fcc(110) 1×2 reconstruction. B) LEED pattern for an fcc(110) c(2x2) reconstruction. The LEED patterns were created using LEEDpat4.[5] C) LEED pattern for clean Au(110) shows the 1×2 reconstruction (E_{beam}=48.0 eV, T=100K). D) LEED pattern for ~0.25 ML of acetate on Au(110) shows a c(2×2) adsorbate ordering on a 1×1 reconstructed surface (E_{beam}=48.0 eV, T=100K). E) LEED pattern for clean Au(110) shows the 1×2 reconstruction (E_{beam}=48.0 eV, T=100K). F) LEED pattern for ~0.25 ML of formate on Au(110) shows a c(2×2) adsorbate ordering on a 1×1 reconstructed surface (E_{beam}=48.0 eV, T=100K). G) LEED pattern for clean Au(110) shows the 1×2 reconstruction (E_{beam}=48.0 eV, T=100K). H) LEED pattern for ~0.25 ML of propanoate on Au(110) shows a c(2×2) adsorbate ordering on a 1×1 reconstructed surface (E_{beam}=48.0 eV, T=100K).

Mild Annealing of a Saturation Coverage of Trifluoroacetate on Au(110)

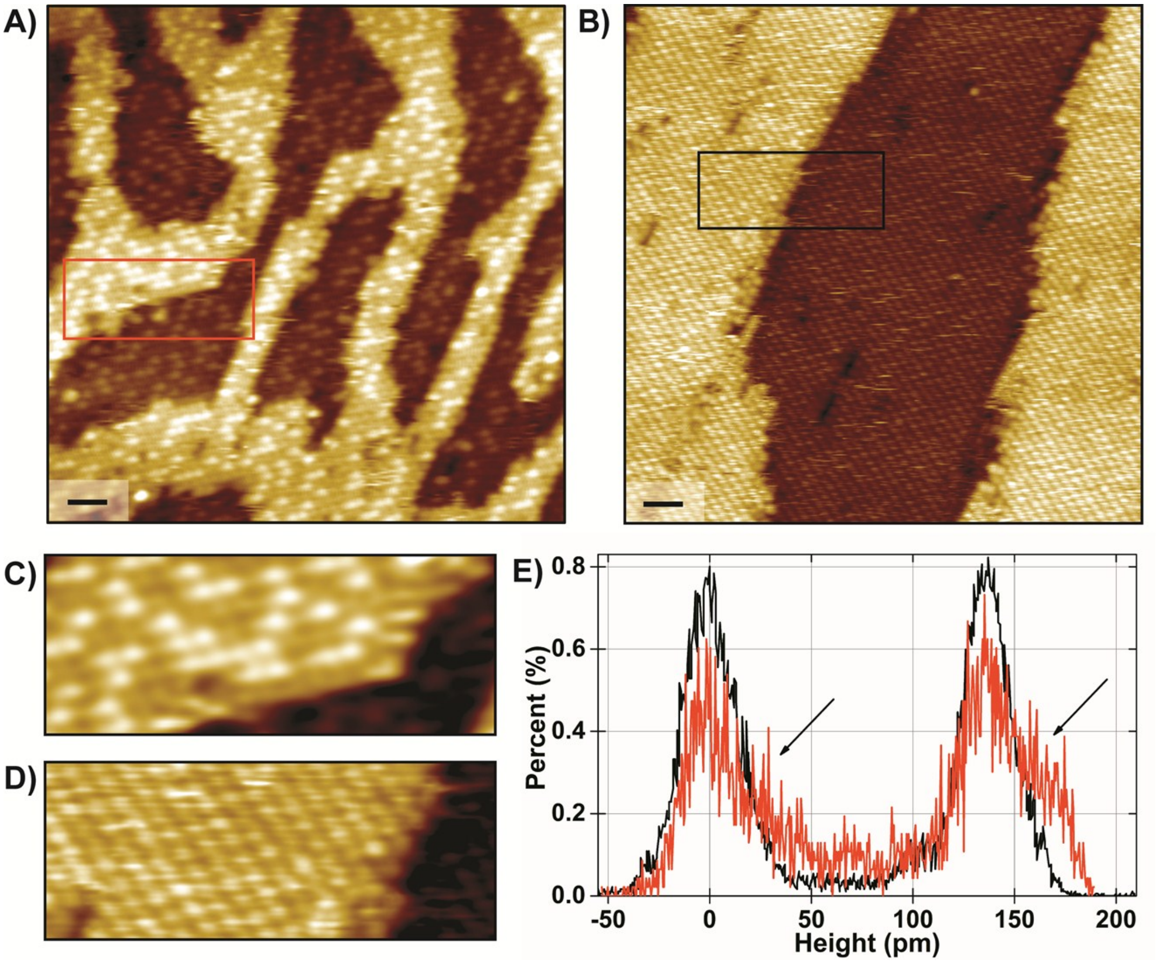

Figure S10: 28 × 28 nm² STM images of the same surface with saturation TFA coverage before (A) and after (B) mild annealing at a temperature below TFA decomposition. Imaging parameters: (A) (+0.5 V ;0.1 nA); (B) (+1.0 V ;0.1 nA); scale bar: 2 nm. (C) A 10 × 3.65 nm² zoom-in image of (A) reveals disordered adsorbate protrusions while (D) a zoom-in image of (B) reveals a homogeneous surface. The homogeneity of the adsorbate protrusions is recovered after annealing and the surface is smoother, without loss in adsorbate coverage. (E) A histogram over

a boxed area of 32 nm^2 on (A) and (B) correspond to red and black lines in (C) and (D). Before annealing, bright protrusions 39 ± 4 pm taller than the background darker protrusions are observed (arrows on red line). After annealing, no bright protrusions are observed, despite a larger corrugation amplitude indicative of a better imaging resolution (black line). Images are processed with the same low-pass FFT filter to subtract high frequency noise. Profiles are averaged over 3 lines.

Interface Energy Calculation

The role of adsorbate-adsorbate interactions on the stability of acetate islands on Au(110) was previously determined using the same methodology detailed here. The change in interface energy from condensation is calculated by comparing the interface energy per carboxylate of 4 super cells containing 1 carboxylate on Au(1×2) as determined according to,

$$\frac{4 \cdot \left[E_{tot}\left(\frac{1}{16}ML_{/Au-1\times2}\right) - n_{Au-1\times2} \cdot \mu_{Au}\right] - 4 \cdot E_{free}}{4} \quad \text{(Eqn. S18)}$$

to the interface energy per carboxylate of 1 super cell with c(2×2) carboxylate layer on Au(1×1) and 3 supercells of the clean Au(1×2) as determined according to,

$$\frac{\left[E_{tot}\left(\frac{1}{4}ML_{/Au-1\times1}\right) - n_{Au-1\times1} \cdot \mu_{Au}\right] + 3 \cdot \left[E_{tot}\left(clean_{/Au-1\times2}\right) - n_{Au-1\times2} \cdot \mu_{Au}\right] - 4 \cdot E_{free}}{4} \quad \text{(Eqn. S19)}$$

where $E_{tot}(clean_{/Au-1\times2})$ is the calculated energy of the supercell of clean Au-1×2 (Fig. S11A), $E_{tot}(1/16ML_{/Au-1\times2})$ is the calculated energy of the supercell of 1/16 ML of carboxylate on Au-1×2 (Fig. S11B), $E_{tot}(1/4ML_{/Au-1\times1})$ is the calculated energy of the supercell of 1/4 ML of carboxylate on Au-1×1 (Fig. S11C), $n_{Au-1\times1}$ is the number of gold atoms in the 1×1 super cell, $n_{Au-1\times2}$ is the number of gold atoms in the 1×2 super cell, μ_{Au} is the chemical potential of a gold atom and E_{free} is the energy of a free (gas phase) carboxylate molecule. The change in interface energy per carboxylate (which quantifies the effect of condensation on carboxylate stability) can be added to the reaction energy per carboxylate (which quantifies the binding strength of isolated carboxylate) calculated previously, for comparing carboxylate stability at the condensed phase.

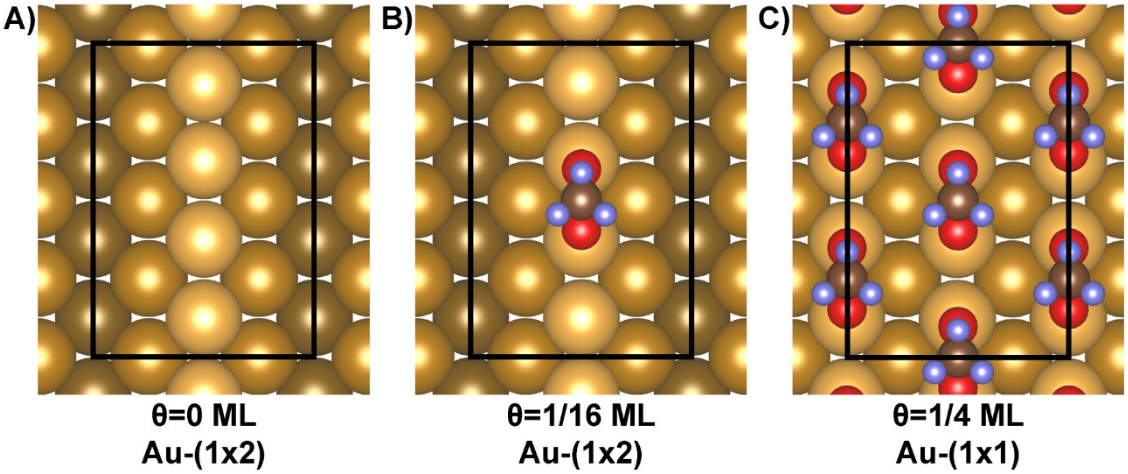

Figure S11. Calculated supercells for (A) clean Au(110)-(1×2), (B) 1/16 ML of trifluoroacetate on Au(110)-(1×2) and (C) 1/4 ML of trifluoroacetate on Au(110)-(1×1). The same geometries were used for all carboxylates.

References

1. Xu, B.; Madix, R. J.; Friend, C. M., Achieving optimum selectivity in oxygen assisted alcohol cross-coupling on gold. *J Am Chem Soc* **2010,** *132* (46), 16571-80.
2. Mozejko, P., Calculations of electron impact ionization cross section for simple biomolecules: Formic and acetic acids. *European Physical Journal-Special Topics* **2007,** *144* (1), 233-237.
3. Gong, J., Structure and surface chemistry of gold-based model catalysts. *Chem Rev* **2012,** *112* (5), 2987-3054.
4. Hiebel, F.; Shong, B.; Chen, W.; Madix, R. J.; Kaxiras, E.; Friend, C. M., Self-assembly of acetate adsorbates drives atomic rearrangement on the Au(110) surface. *Nat Commun* **2016,** *7*, 13139.

5. "The software utility LEEDpat, Version 4.2, (C) K.E. Hermann (FHI Berlin) and M.A. Van Hove (Baptist University Hong Kong) was used in the evaluation of the data; see also http://www.fhi-berlin.mpg.de/KHsoftware/LEEDpat/index.html."
